# Molecular hydrogen stimulates the gene expression of transcriptional coactivator PGC-1α to enhance fatty acid metabolism

**DOI:** 10.1038/npjamd.2016.8

**Published:** 2016-04-28

**Authors:** Naomi Kamimura, Harumi Ichimiya, Katsuya Iuchi, Shigeo Ohta

**Affiliations:** 1 Department of Biochemistry and Cell Biology, Institute of Development and Aging Sciences, Graduate School of Medicine, Nippon Medical School, Kawasaki-city, Japan; 2 Department of Neuroregenerative Medicine, Juntendo University Graduate School of Medicine, Tokyo, Japan

## Abstract

We previously reported that molecular hydrogen (H_2_) acts as a novel antioxidant to exhibit multiple functions. Moreover, long-term drinking of H_2_-water (water infused with H_2_) enhanced energy expenditure to improve obesity and diabetes in *db*/*db* mice accompanied by the increased expression of fibroblast growth factor 21 (FGF21) by an unknown mechanism. H_2_ was ingested by drinking of H_2_-water or by oral administration of an H_2_-producing material, MgH_2_. The comprehensive gene expression profile in the liver of *db*/*db* mice was analyzed by DNA microarray. The molecular mechanisms underlying the gene expression profile was investigated using cultured HepG2 cells. Moreover, the effects on lifespan of drinking H_2_-water were examined using wild-type mice that were fed a fatty diet. Pathway analyses based on comprehensive gene expression revealed the increased expression of various genes involved in fatty acid and steroid metabolism. As a transcription pathway, the PPARα signaling pathway was identified to upregulate their genes by ingesting H_2_. As an early event, the gene expression of PGC-1α was transiently increased, followed by increased expression of *FGF21*. The expression of *PGC-1α* might be regulated indirectly through sequential regulation by H_2_, 4-hydroxy-2-nonenal, and Akt/FoxO1 signaling, as suggested in cultured cell experiments. In wild-type mice fed the fatty diet, H_2_-water improved the level of plasma triglycerides and extended their average of lifespan. H_2_ induces expression of the *PGC-1α* gene, followed by stimulation of the PPARα pathway that regulates FGF21, and the fatty acid and steroid metabolism.

## Introduction

We previously reported that molecular hydrogen (H_2_) acts as a novel antioxidant and effectively protects cells against oxidative stress.^1^ Subsequently, it was revealed that H_2_ exhibits multiple functions, including anti-inflammation, anti-apoptosis, anti-allergy and regulation of differentiation, in addition to anti-oxidative functions.^2,3^ Many publications have strongly suggested that H_2_ has potential for broad therapeutic and preventive applications because of its lack of adverse effects.^3^ In addition to extensive animal experiments, >10 papers on clinical studies have been published, including on double-blinded clinical studies for patients with Parkinson’s disease and rheumatism.^4,5^ The field of hydrogen medicine is highly expected to deliver actual medical applications in many diseases.

In addition to anti-oxidative roles, we reported the benefit of *ad libitum* drinking of H_2_-water (water infused with H_2_) for type 2 diabetes using *db/db* obesity model mice that lack the functional leptin receptor.^6^ Long-term drinking of H_2_-water significantly decreased body and fat weights, and the levels of plasma glucose, insulin, and triglyceride. Importantly, the *db*/*db* mice ingested the same amounts of water and diet. Moreover, we found enhanced expression of a hepatic hormone, fibroblast growth factor 21 (FGF21), which is known to function to enhance fatty acid and glucose expenditure.^6^ On the other hand, the phosphoenolpyruvate carboxykinase (PEPCK) and glucose-6-phosphatase, catalytic subunit (G6PC) genes involved in gluconeogenesis were not affected.^6^ These results suggest the potential benefit of H_2_ in improving obesity, diabetes, and metabolic syndrome. In fact, drinking H_2_-water improved nonalcoholic steatohepatitis (NASH) model mice^7^ and a clinical study indicated that it caused a decrease in low-density lipoprotein in patients with metabolic syndrome.^8^

To understand the molecular mechanism by which H_2_ stimulates energy metabolism, it needs to be clarified whether long-term drinking of H_2_-water (e.g., for 3 months) primarily regulates gene expression to exhibit phenotypic change or conversely phenotypic changes influence gene expression as a secondary consequence.

To reveal the causal association among drinking H_2_-water, gene expression and phenotypes, we comprehensively analyzed time-dependent expression by microarray, and found that H_2_ stimulates the gene expression of a transcriptional coactivator, peroxisome proliferator-activated receptor-γ coactivator-1α (PGC-1α), as an early event, followed by activation of the peroxisome proliferator-activated receptor α (PPARα) pathway to transcribe the genes involved in fatty acid metabolism. The expression of *PGC-1α* might be regulated indirectly through sequential regulation by H_2_, 4-hydroxy-2-nonenal (4-HNE), and the Akt (or Protein Kinase B (PKB))/Forkhead box protein O1 (FoxO1) signaling. In addition, we show that drinking H_2_-water improved plasma triglycerides and extended the average of lifespan of the wild-type mice that were fed a fatty diet.

## Results

### Long-term consumption of H_2_-water increased the expression of various hepatic metabolic genes

To clarify the causal association in drinking H_2_-water between gene expression and stimulated energy metabolism, we attempted to identify the changes in gene expression at the early stage before a phenotype appears. When H_2_-water was given for 14 days, no significant phenotype was observed as judged by body weight and the plasma levels of glucose and triglyceride (Supplementary Figure S1). Thus, we comprehensively screened all genes by DNA microarray on day 14 in order to explore candidate genes that induce the appearance of a phenotype. The hepatic gene expression profiles in mice drinking both H_2_-water and degassed version as control water were examined using Agilent cDNA microarray technology. Analysis was performed on three samples in each group to evaluate the statistical significance of differences.

**Figure 1:**
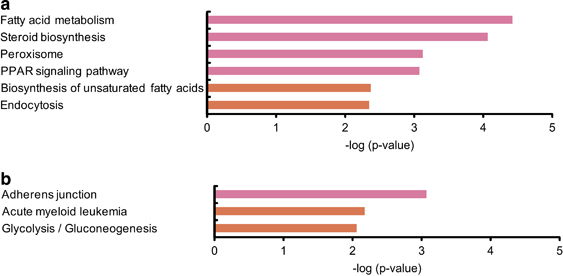
Microarray-based pathway analysis of genes affected by 14 days of consumption of H_2_. Top-ranked pathways involving (**a**) upregulated and (**b**) downregulated genes by drinking H_2_-water for 14 days. *Db*/*db* mice were given water with or without H_2_ for 2 weeks. Total RNA was prepared from the liver and DNA microarray analysis was performed. All microarray data were subjected to the KEGG (Kyoto Encyclopedia of Genes and Genomes) pathway analysis. The results are expressed as −log (*P* value). Pink bar, *P*<0.001; orange bar, *P*<0.01.

A total of 1,886 genes were significantly differentially expressed, including 1,344 upregulated genes and 542 downregulated ones shown as a heat map panel (Supplementary Figure S2); however, there were no genes for which the expression level changed more than twofold in the H_2_-water group and that belong to the Kyoto Encyclopedia of Genes and Genomes (KEGG) pathway DataBase, suggesting that the effects of H_2_ are mild. As the change in the expression of each gene was small but significant, the candidate genes were explored using KEGG pathway analysis. For the analysis on the upregulated genes, six KEGG pathways were found to be significantly changed (*P*<0.01; Figure 1a). Among these pathways, four pathways were highly significant at *P*<0.001. For the analysis on the downregulated genes, three KEGG pathways were found to be significantly changed (*P*<0.01; Figure 1b).

**Figure 2:**
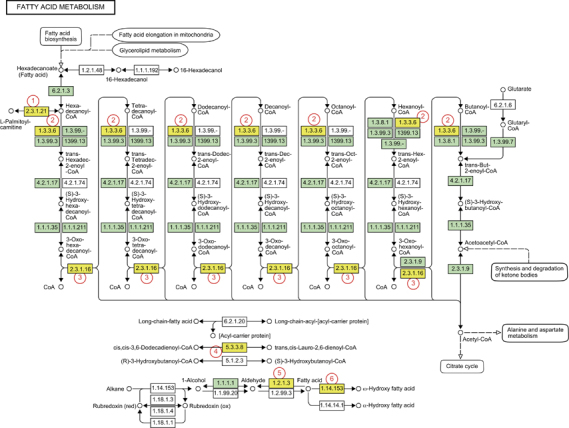
Hydrogen enhances the expression of a wide variety of fatty acid metabolism-related genes. Differentially expressed genes identified in the KEGG pathway database related to the fatty acid metabolic pathway are shown. Genes for which the expression was significantly increased by H_2_-water in the pathway are indicated in yellow: (1) carnitine palmitoyltransferase 1a, liver (*Cpt1a*); (2) acyl-coenzyme A oxidase 1, palmitoyl (*Acox1*); (3) acetyl-coenzyme A acyltransferase (*Acaa1a, Acaa1b*); (4) dodecenoyl-coenzyme A delta isomerase (3, 2-trans-enoyl-coenyme A isomerase) (*Dci*), peroxisomal delta3, delta2-enoyl-coenzyme A isomerase (*Peci*); (5) aldehyde dehydrogenase (*Aldh1a1*, *Aldh3a2*, *Aldh1l1*); (6) cytochrome P450, family 4, subfamily a, polypeptide (*Cyp4a32*, *Cyp4a31, Cyp4a10*).

Among these pathways, fatty acid metabolism was identified as only one pathway changed at *P*<0.0001. Reverse transcription PCR analysis confirmed the significantly increased expression of genes that are involved in fatty acid metabolism (Supplementary Figure S3). The relationships among genes involved in fatty acid metabolism, steroid biosynthesis, peroxisome, and the PPAR signaling pathway are illustrated according to the KEGG pathway (Figure 2, Supplementary Figures S4–S6, respectively). Although the genes sodecenoyl-coenzyme A delta isomerase (*Dci*) and aldehyde dehydrogenase (*Aldh3aa2*) associated with fatty acid metabolism are not currently classified as members of the PPARα pathway in the KEGG DataBase, they are target genes of PPARα, as described in a previous report.^9^ In addition, the PPARα pathway is known to regulate steroid metabolism as well as fatty acid metabolism.^9,10^ Thus, we focus on the PPARα pathway as the early event that H_2_ causes.

### Consumption of H_2_-water induces hepatic *PGC-1α* gene expression

As shown above, H_2_ influences gene expression upon 2 weeks of its consumption. We also investigated the effect of H_2_ for shorter periods. Although an amount of H_2_ in H_2_-water is limited, MgH_2_ can produce a desired quantity of H_2_ by the following reaction in the stomach.

$${\mathrm{MgH}}_{2}+{\mathrm{2H}}_{2}O\to \mathrm{Mg}{\left(\mathrm{OH}\right)}_{2}+{\mathrm{2H}}_{2}$$

**Figure 3:**
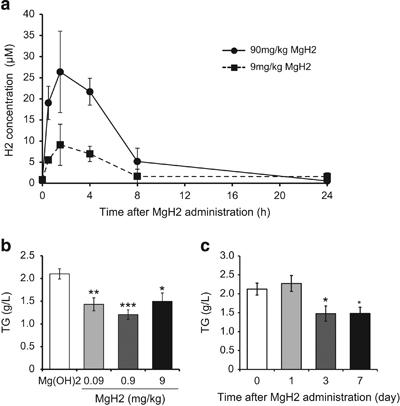
H_2_ is detected in blood after oral administration of MgH_2_ and reduced plasma triglyceride level of db/db mice. (**a**) Rats were administered MgH_2_ suspension orally by stomach gavage at 9 mg/kg or 90 mg/kg. After 0.5, 1.5, 4, 8, and 24 h, hydrogen concentration in blood was quantified using gas chromatography, as described in Materials and Methods. (**b**) *Db*/*db* mice were given 0.09, 0.9 or 9 mg/kg MgH_2_ for 28 days. An equal amount of Mg(OH)_2_, which was produced by 9 mg/kg MgH_2_, was used as a control. Plasma concentrations of triglycerides are shown as mean±s.e.m. (*n*=15). *P<0.05, **P<0.01, ***P<0.001. (**c**) *Db/db* mice were given 0.9 mg/kg MgH_2_ for 0, 1, 3, and 7 days. Plasma concentrations of triglycerides are shown as mean±s.e.m. (*n*=15). *P<0.05.

When rats can be orally given MgH_2_, blood H_2_ slowly increased in a dose-dependent manner (Figure 3a). When mice were given MgH_2_ once a day for 4 weeks, the level of plasma triglyceride decreased at the maximum dose of 0.9 mg/kg (Figure 3b). Mg(OH)_2_ was administered exactly in the same way as a control to avoid the effects of any extrinsic factors. An effect on plasma triglycerides was observed by a single administration per day for 3 days (Figure 3c).

**Figure 4:**
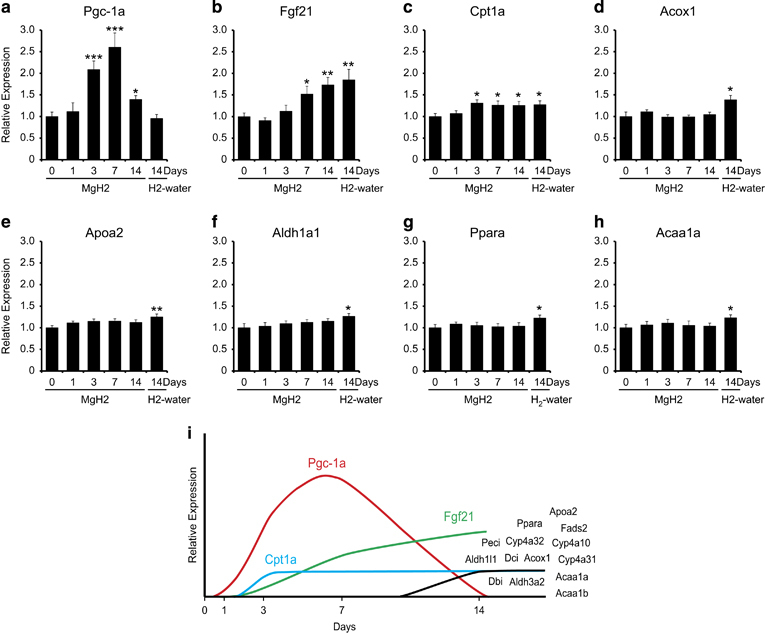
Three days of consumption of H_2_-water induces hepatic *PGC-1α* gene expression. (**a**–**h**) Quantitative RT-PCR analysis was used to confirm the differential expression of genes identified by microarray and pathway analyses. A total of eight genes were selected for primer design. The effects of consuming hydrogen for 1–14 days on hepatic gene expression in *db*/*db* mice were analyzed. Data are mean±s.e.m. *P<0.05, **P<0.01, ***P<0.001, versus 0 days (*n*=9). (**i**) Scheme of gene expression pattern affected by H_2_. RT-PCR, Reverse transcription PCR.

We performed microarray analysis to examine gene expression change by 1, 3, and 7 days of administration of MgH_2_. One day after administration, there was no significant change in gene expression among the genes selected by the result of 2-week H_2_-water consumption (Supplementary Figure S7). Whole-genome analysis by microarray showed that *PGC-1α* expression in *db*/*db* mice increased markedly upon 3 days and 7 days of administration, which was confirmed by reverse transcription PCR (Figure 4a). After 3 days of administration of MgH_2_, carnitine palmitoyltransferase 1A (*Cpt1a*) gene expression increased, and after 7 days of administration, *FGF21* gene expression increased significantly (Figure 4b, c). In contrast, the expression of *PGC-1α* in C57BL/6 wild-type was not affected by the oral administration of MgH_2_ (Supplementary Figure S8). This result suggested that the expression *PGC-1α* is stimulated by H_2_ only in some pathogenic status.

### The expression of *PGC-1α* is regulated sequentially through H_2_, 4-HNE, Akt, and FoxO1

**Figure 5:**
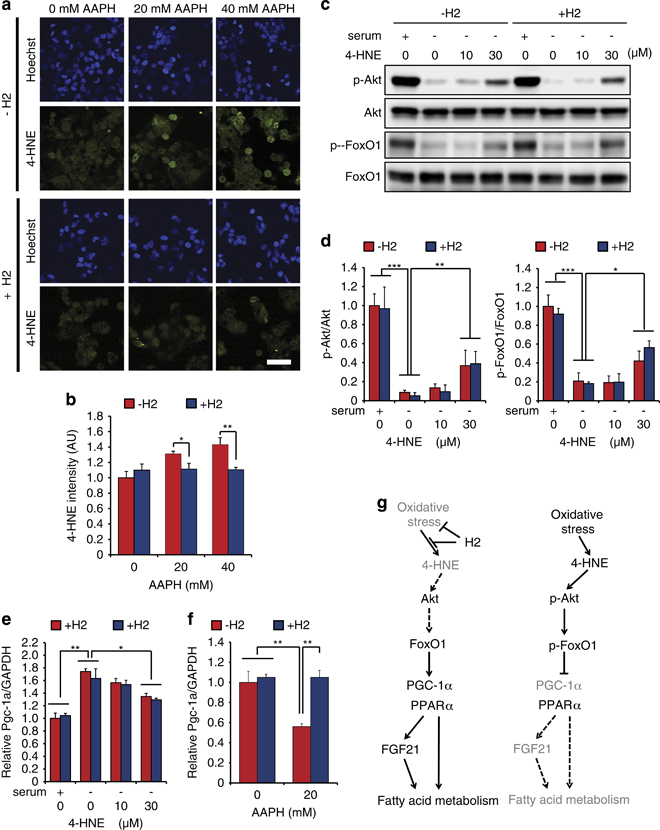
Effects of H_2_ on 4-HNE production and 4-HNE-induced Akt/FoxO1 signaling. (**a**, **b**) Effect of H_2_ on 4-HNE generation. HepG2 cells were treated with AAPH at the indicated concentrations for 6 h in the absence or presence of 10% H_2_. (**a**) Representative images of 4-HNE immunostaining (Scale bar: 50 μm). (**b**) 4-HNE staining was semi-quantified. *P<0.05 vs. **P<0.01 vs. no H_2_ (*n*=3) (**c**, **d**) Effects of 4-HNE on Akt/FoxO1 signaling. Cells were treated with 4-HNE at the indicated concentrations for 1 h with (+serum) or without (−serum) of 10% fetal bovine serum in the presence (+H_2_) or absence (−H_2_) of 50% H_2_. Cells were lysed, and phosphorylation of Akt and FoxO1 was analyzed by western blotting. (**c**) Representative western blot of three independent experiments. (**d**) Quantification of the blots. The amounts of phosphorylated proteins were normalized with the corresponding total protein. Data are shown as mean±s.d. (*n*=3). *P<0.05, **P<0.01 and ***P<0.001. (**e**) Effects of H_2_ on 4-HNE-induced suppression of *PGC-1α* gene expression. Cells were treated with 4-HNE at the indicated concentrations for 6 h in the presence or absence of 50% H_2_ under a serum-free condition. Total RNA was prepared from the cells, and the expression of the *PGC-1α* gene level was estimated using quantitative RT-PCR analysis. Data are mean±s.d. (*n*=3). *P<0.05 and **P<0.01. (**f**) HepG2 was exposed to AAPH for 6 h in the absence (−H_2_) or presence (+H_2_) of 10% H_2_, and then the expression of the *PGC-1α* gene level was estimated as described above. Data are mean±s.d. (*n*=3). **P<0.01. (**g**) A hypothetical model of effects of H_2_. H_2_ suppresses oxidative stress, especially the generation of lipid peroxides and their end products including 4-HNE. The phosphorylation of Akt, followed by the phosphorylation of FoxO1 (a transcription factor of the *PGC-1α* gene), is recovered by 4-HNE. Thus, H_2_ indirectly suppresses the phosphorylations of Akt and FoxO1 through suppressing 4-HNE generation. Because the phosphorylated FoxO1 is an inactive form, H_2_ indirectly recoveres the expression of the *PGC-1α* gene. The recovered *PGC-1α* expression leads to the PPARα pathway that upregulates FGF21, and fatty acid and steroid metabolism. AAPH, 2,2′-azobis(2-amidinopropane) dihydrochloride; RT-PCR, reverse transcription PCR.

Next, the molecular pathway to upregulate *PGC-1α* was investigated using a hepatocyte-derived cultured cell line (HepG2). We previously reported that H_2_ reduces cellular hydroxyl radicals, which is a trigger of the free-radical chain reaction, so it should prevent the free-radical chain reaction, resulting in decreases in peroxides and their end products including 4-HNE.^1,11,12^ Growing evidence suggests specific functions of 4-HNE as a second messenger in oxidative stress signaling.^13,14^ Moreover, oxidative stress is increased by obesity.^15,16^ Thus, we speculated that 4-HNE is initially involved in the pathway. Indeed, we demonstrated that H_2_ decreased 4-HNE in the presence of a free-radical inducer, 2,2′-azobis(2-amidinopropane) dihydrochloride in HepG2 cells (Figure 5a, b).

Because *PGC-1α* is transcribed by transcription factors, forkhead box protein O1 (FoxO1) and cAMP-response element-binding protein, using each of their dependent promoters,^17^ and FoxO1 is phosphorylated by phosphorylated PKB or Akt, causing nuclear exclusion, resulting in suppression of the expression of *PGC-1α*.^17^ We found that 4-HNE recovered the phosphorylations of Akt and FoxO1 under a serum-free condition (Figure 5c, d); however, H_2_ did not affect these phosphorylations (Figure 5c, d). In contrast, 4-HNE downregulated *PGC-1α* expression (Figure 5e). Importantly, H_2_ did not directly affect the regulation of *PGC-1α* in the presence or absence of 4-HNE (Figure 5e); however, H_2_ recovered the *PGC-1α* expression that was suppressed by 2,2′-azobis(2-amidinopropane) dihydrochloride (Figure 5f). Thus, the expression of *PGC-1α* is possibly regulated by the pathway sequentially though H_2_, 4-HNE, the phosphorylation of Akt and FoxO1. The speculative molecular mechanism is illustrated in Figure 5g based on these experiments using HepG2 cells.

### Drinking H_2_-water improves triglycerides and lifespan

**Figure 6:**
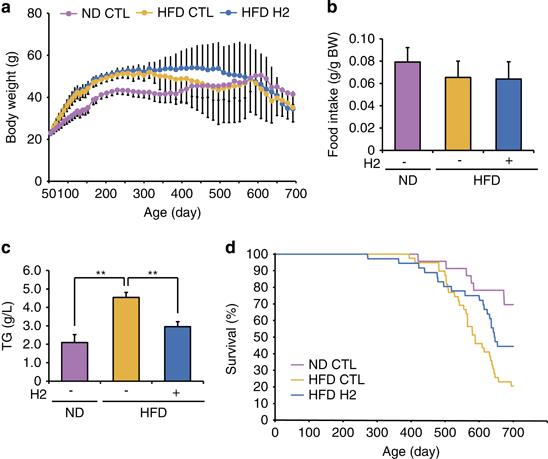
Consuming H_2_-water reduces plasma triglyceride levels and extends lifespan in DIO (diet-induced obesity) mice. C57BL/6 mice were fed a normal diet (ND) or a high-fat diet (HFD) with or without H_2_-water for 74 weeks. (**a**) Body weights of mice were measured every 2 weeks throughout the experiment. Data are mean±s.d. (**b**) Average food intakes per body weight are shown. Data are mean±s.d. (**c**) Plasma triglyceride concentrations are shown. Data are mean±s.e.m. **P<0.01 (*n*=4–6). (b) Survival curves for C57BL/6 mice fed ND or HFD with or without H_2_-water. Lifespan was increased (*P*=0.032) in the H_2_-consuming HFD group compared with that in the control HFD group (ND group: *n*=23, HFD control water group: *n*=38, HFD H_2_-water group: *n*=36). (**c**) Body weights of mice were measured every 2 weeks throughout the experiment. Data are mean±s.d. (**d**) Average food intakes per body weight are shown. Data are mean±s.d.

Finally, we examined the effects of the H_2_-water in wild-type mice that were fed a fatty diet instead of *db*/*db* mice. H_2_-water did not affect body weight and food intake (Figure 6a, b); however, drinking H_2_-water decreased the plasma level of triglycerides and increased the average of lifespan (Figure 6c, d). Thus, drinking H_2_-water could be beneficial for wild-type mice fed with a fatty diet.

## Discussion

Our previous study indicated that 3 months of consumption of H_2_-water improved obesity (body-fat weight) and diabetes (glucose, insulin, and triglycerides) in *db*/*db* mice accompanied by increased expression of the *FGF21* gene; however, the causal association among their improved phenotypes, stimulated energy metabolism, and gene expression was unclear because of their long-term mutual interactions. In the present study, we comprehensively examined the temporal changes in hepatic gene expression in *db*/*db* mice that had ingested H_2_. Although H_2_ did not strongly influence each gene’s expression, the KEGG pathway analysis of microarray data revealed with strong significance that genes involved in fatty acid and steroid metabolism were expressed through the PPARα signaling pathway.

For further analysis in shorter administration periods, we used MgH_2_, which produces H_2_ in the stomach. The ingestion of H_2_ for 3 days induced the gene expression of *PGC-1α*, accompanied by a decrease in plasma triglyceride, and followed by an increase in *FGF21*.

PGC-1α, FGF21, and PPARα are very important regulators of energy metabolism. PGC-1α is a member of a family of transcription coactivators that have a central role by activating various transcription factors in the regulation of cellular energy metabolism.^18,19^ When PGC-1α activates the transcription factor PPARα, fatty acid metabolism is enhanced.

The PPARs are members of a relatively large family of nuclear receptors and function as ligand-activated transcriptional factors, all of which are subject to transcriptional coactivation by PGC-1α. PPARα regulates the expression of genes involved in fatty acid β-oxidation.^9,20^

FGF21 is strongly induced in liver by prolonged fasting via PPAR-α.^21^ FGF21 stimulates the phosphorylation of fibroblast growth factor receptor substrate 2 and extracellular signal-regulated protein kinases 1 and 2 (ERK1/2) to induce the hepatic expression of key regulators of gluconeogenesis, lipid metabolism, and ketogenesis.^22^

At the early stage, carnitine palmitoyltransferase 1α (Cpt-1α) slightly but significantly increased. Cpt-1α is transcribed by transcription factors PPARα and TR-β, both of which are coactivated by PGC-1α.^23^ Thus, it is likely that the expression of *Cpt-1α* was enhanced by PGC-1α.

The interactions among these key factors are complicated: *FGF21* is *PGC-1α* dependently transcribed by PPARα, while FGF21 induces *PGC-1α*.^21,24,25^ Although there are complicated interactions among the key factors, we found that H_2_ increases the gene expression of *PGC-1α* as the early event. Thus, PGC-1α activates PPARα, resulting in stimulation of the PPARα pathway. PPARα transcribes the *FGF21* gene and genes involved in fatty acid metabolism and steroid metabolism. In turn, FGF21 stimulates the expression of fatty acid metabolism as a hormonal function, as illustrated in Figure 5g.

H_2_ reduces hydroxyl radicals, which is a trigger of the free-radical chain reaction, so it should prevent the free-radical chain reaction, resulting in decreases of peroxides and their end products including 4-HNE.^1^ In this study, we found H_2_ decreased 4-HNE when free radicals were induced (Figure 5a, b), which agreed with previous studies.^1,11,12^

*PGC-1α* is transcribed by transcription factors FoxO1 and cAMP-response element-binding protein, using each of their dependent promoters.^17^ FoxO1 is phosphorylated by activated Akt, causing nuclear exclusion, resulting in suppression of the expression of *PGC-1α*.^17^ Indeed, the phosphorylation of Akt and FoxO1 was recovered by 4-HNE, but not by H_2_, under a serum-free condition in HepG2 cells. Thus, although these conditions in HepG2 cells were far from the physiological conditions in *db*/*db* mice, we speculated the involvement of 4-HNE and the phosphorylation of Akt and FoxO1 in inducing *PGC-1α*, as illustrated in Figure 5g.

PGC-1α also functions as the organizer of mitochondrial biogenesis. Recently, it was reported that H_2_ enhances mitochondrial membrane potential in damaged sperm.^26^ Multiple functions of H_2_ may be elucidated at least partly through the multiple functions of PGC-1α.

Finally, we showed that prolonged drinking of H_2_-water improved the plasma triglyceride level and extended the average of lifespan in wild-type mice that were fed a fatty diet. It is possible that the lifespan-extending effect of H_2_-water can be not only based on the effect on the liver but also on skeletal muscle or other organs. Because FGF21 was previously reported to increases lifespan,^27^ increased energy metabolism in the liver could be one of the major contributors for the extension of the average of lifespan.

## Materials and methods

### Animals

This study was approved by the Animal Care and Use Committee of Nippon Medical School (Tokyo, Japan). The methods were carried out in ‘accordance’ with the relevant guidelines and regulations.

Genetically diabetic male *db*/*db* mice (BKS.Cg-+*Leprdb*/+*Leprdb*/Jcl) and their non-diabetic heterozygous *db*/*+* littermates (BKS.Cg-m+/+*Leprdb*/Jcl) were purchased at 5 weeks of age from CLEA Japan (Tokyo, Japan). For the diet-induced obesity study, C57BL/6 mice were given a high-fat diet (D12451; RESEARCH DIETS, New Brunswick, NJ, USA). Normal-fat diet (D12450B; RESEARCH DIETS) was used for the control group. C57BL/6 mice of 12 weeks of age and male Sprague-Dawley rats of 10 weeks of age were purchased from Nippon SLC (Hamamatsu, Shizuoka, Japan).

### Hydrogen treatment

Water with dissolved molecular hydrogen (H_2_-water) was used for 2 weeks of consumption. The H_2_-water was prepared as described previously.^11^ Mice were given water freely using closed glass vessels equipped with an outlet line containing two ball bearings, which kept the water from being degassed. The vessel was refilled with fresh H_2_-water every day. H_2_-water degassed by gentle stirring was used as control water.

MgH_2_, which reacts with H_2_O and produces H_2_, was used for short term consumption. MgH_2_ powder was suspended in glycerol that had been dehydrated with molecular sieves to prevent MgH_2_ from reacting with H_2_O. Mice received MgH_2_ suspension orally by stomach gavage at 9 or 90 mg/kg once a day. MgH_2_ reacted with H_2_O in the stomach to produce H_2_. Mg(OH)_2_ was used as a control. Hydrogen concentration in blood was measured as described previously.^1^

### Microarray analysis

Total RNA was isolated separately from each mouse tissue using an RNeasy Mini kit (QIAGEN, Valencia, CA, USA) according to the manufacturer’s instructions and dissolved in RNase-free water at a final concentration of 2.0 μg/μl. Nine RNA samples were divided into three sets and made into mixtures (each set contained RNA from three mice). These three RNA sample sets of each experimental group were used for microarray analysis.

Total RNA was labeled using a Low-Input QuickAmp Labeling Kit, Two-Color (Agilent Technologies, Santa Clara, CA, USA). Cy3 dye was used to label cDNA from the control-water group and Cy5 dye was used to label cDNA from the H_2_-water group. Gene expression analysis was performed on three independent samples for each group using a microarray (SurePrint G3 Mouse GE 8×60 K v2 Microarray, Agilent Technologies, Santa Clara, CA, USA). To compare the results of the three sets of microarray experiments, the signal intensity of each gene from different arrays was normalized by the total intensity in each array. Signal evaluation was performed using Agilent Feature Extraction Software (Agilent Technologies). Statistical analysis was applied to select the differentially expressed genes. Only cases with signal evaluation score=2, and *P* value <0.05 were identified as differentially expressed genes. For the expression assay for *db*/*+* and *db*/*db* mice, Cy3 and Cy5 dyes were used, respectively.

The raw microarray data were deposited in the Gene Expression Omnibus (accession number, GSE71738; http://www.ncbi.nlm.nih.gov/geo/query/acc.cgi?acc=GSE71738).

### KEGG pathway analysis

A pathway enrichment analysis of differentially expressed genes was conducted using KEGG pathway information (http://www.genome.jp/kegg/pathway.html). Probe set IDs of each category were first mapped to NCBI Entrez gene IDs according to the Agilent Mouse Array annotation file, and then were mapped to KEGG gene IDs according to the KEGG gene cross-reference file. Pathways that were significantly enriched with differentially expressed genes were identified. Graphical pathway maps were downloaded from the KEGG site, and differentially expressed genes were then highlighted in yellow.

### Quantitative real-time RT-PCR (q-PCR)

Complementary DNA was generated by SuperScript II Reverse Transcriptase (Thermo Fisher Scientific Inc., Waltham, MA, USA) from RNA samples that were used for microarray analysis. cDNA was analyzed by quantitative PCR using Thermal Cycler Dice Real Time System TP800 (TAKARA BIO INC., Otsu, Shiga, Japan). All samples were normalized to glyceraldehyde 3-phosphate dehydrogenase (GAPDH) expression. Primer and probe sequences for each PCR are shown in Supplementary Table S1.

### Cell culture and treatment of cells

HepG2 human hepatoma cells were treated with 2,2′-azobis(2-amidinopropane) dihydrochloride for 6 h in the presence or absence of 10% H_2_. For 4-HNE treatment, cells were treated with 4-HNE for 1 h for analysis of phosphorylation or 6 h for analysis of gene expression in the presence or absence of 50% H_2_.

### Immunofluorescence and western blotting

Immunofluorescence was examined as described previously^1^ using anti-4-HNE antibodies (3.3 μg/ml; MHN-100P; Japan Institute for the Control of Aging, Japan). The cells were counterstained with Hoechst 33342. Immunofluorescence was observed using a laser scanning confocal microscope (FV1200; Olympus, Tokyo, Japan).

For western blotting, the following primary antibodies were used in 5% BSA in TBST: anti-p-Akt (1:5,000, #4060: Cell Signaling Technology, Danvers, MA, USA), anti-total Akt (1:1000, #4691: Cell Signaling Technology), anti-p-FoxO1 (1:2500, #2599: Cell Signaling Technology), anti-total FoxO1 (1:1000, #2880: Cell Signaling Technology). Horseradish peroxidase-conjugated anti-rabbit IgG antibody (sc-2004: Santa Cruz Biotechnology, Dallas, TX, USA) was used as a secondary antibody.

### Measurement of plasma triglyceride

Plasma concentrations of triglyceride were determined with commercially available kits (Wako Pure Chemical Industries, Osaka, Japan).

### Statistical analysis

We performed statistical analysis by applying an unpaired two-tailed Student's *t*-test, as described previously.^1^ Differences were considered statistically significant at *P*<0.05.
