# Supplementary material for: Molecular hydrogen stimulates the gene expression of transcriptional coactivator PGC-1α to enhance fatty acid metabolism
Source: NPJ Aging Mech Dis. 2016 Apr 28;2:16008–. doi: 10.1038/npjamd.2016.8 (PMC5515010; doi:10.1038/npjamd.2016.8)
Supplement: Supplementary Information [file npjamd20168-s1.pdf]

Supplementary Figure S1

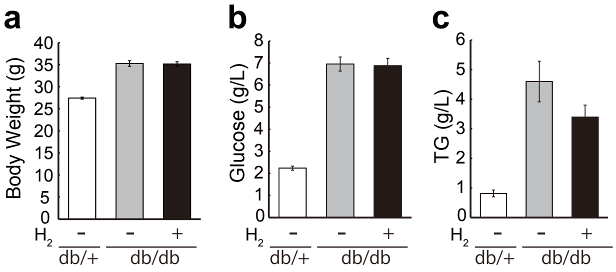

Supplementary Figure S1. Consumption of H_2-water for 14 days shows no significant effects on body weight and the plasma levels of glucose and triglycerides.

(**a**) Body weights of *db/db* mice given water with or without hydrogen for 14 days were measured. Data are mean ± SEM (n = 9). *Db/+* mice were used as normal mouse controls. (**b**) Plasma concentrations of glucose and (**c**) triglycerides are shown as mean ± SEM (n = 9 for each *db/db* mice group and n=6 for *db/+* mice group).

Supplementary Figure S2

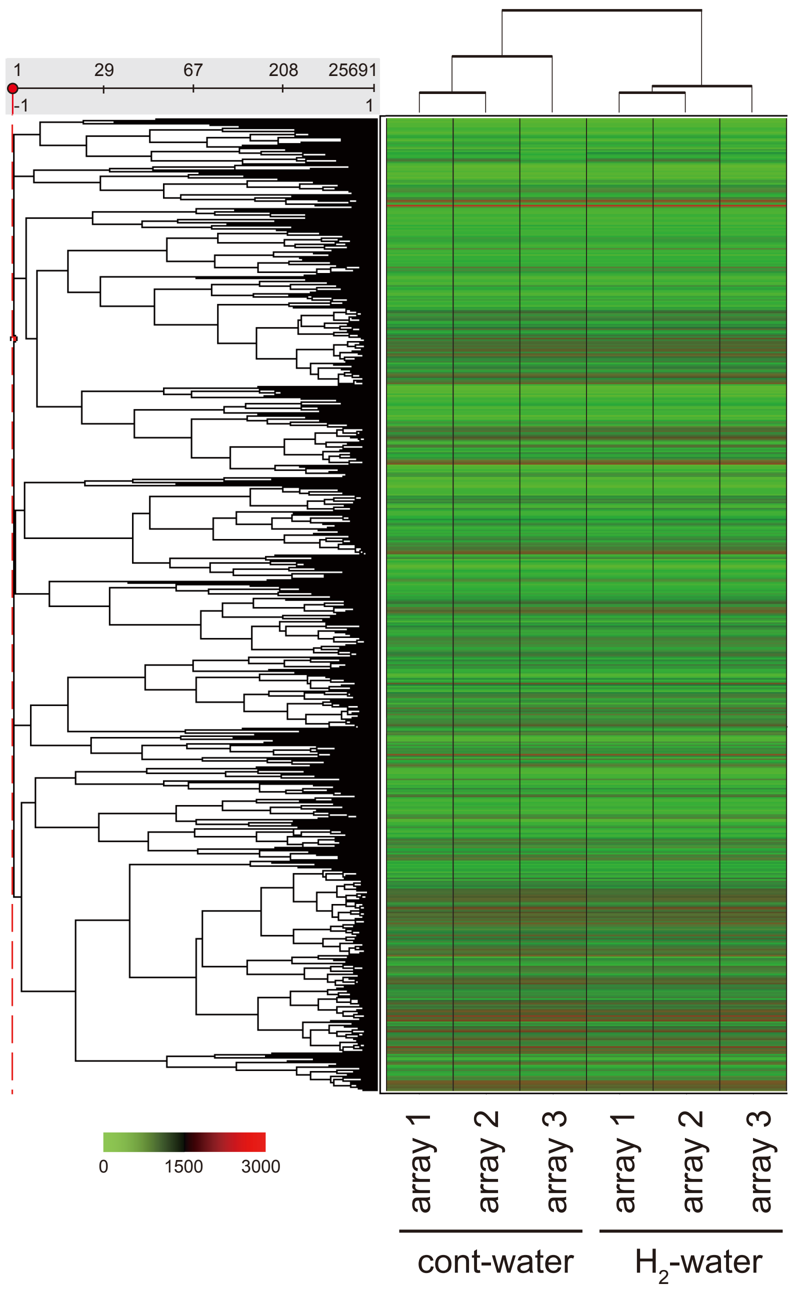

Supplementary Figure S2. Heat map representing the expression of hepatic genes of *db/db* mice given water with or without hydrogen for 14 days.

Colors represent expression levels of each gene.

Supplementary Figure S3

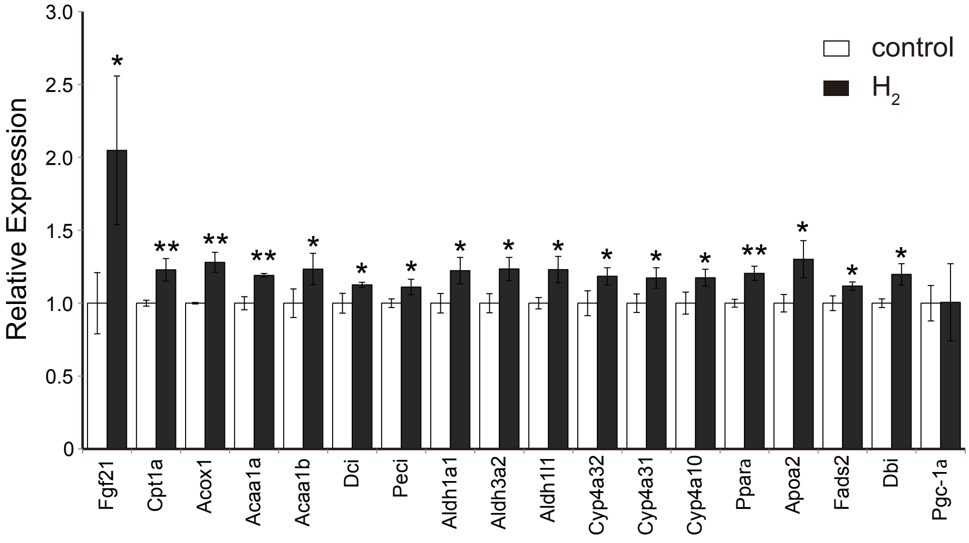

Supplementary Figure 3. Relative expression on DNA microarray of genes up-regulated by H_2-water for 2 weeks.

Db/db mice were given water with or without H_2 for 2 weeks. Total RNA was prepared from the liver and DNA microarray analysis was performed. All microarray data were submitted to the KEGG (Kyoto Encyclopedia of Genes and Genomes) pathway analysis. Relative signal intensities of genes selected by the pathway analysis are shown. Data are mean ± SD (n=3).

Supplementary Figure S4

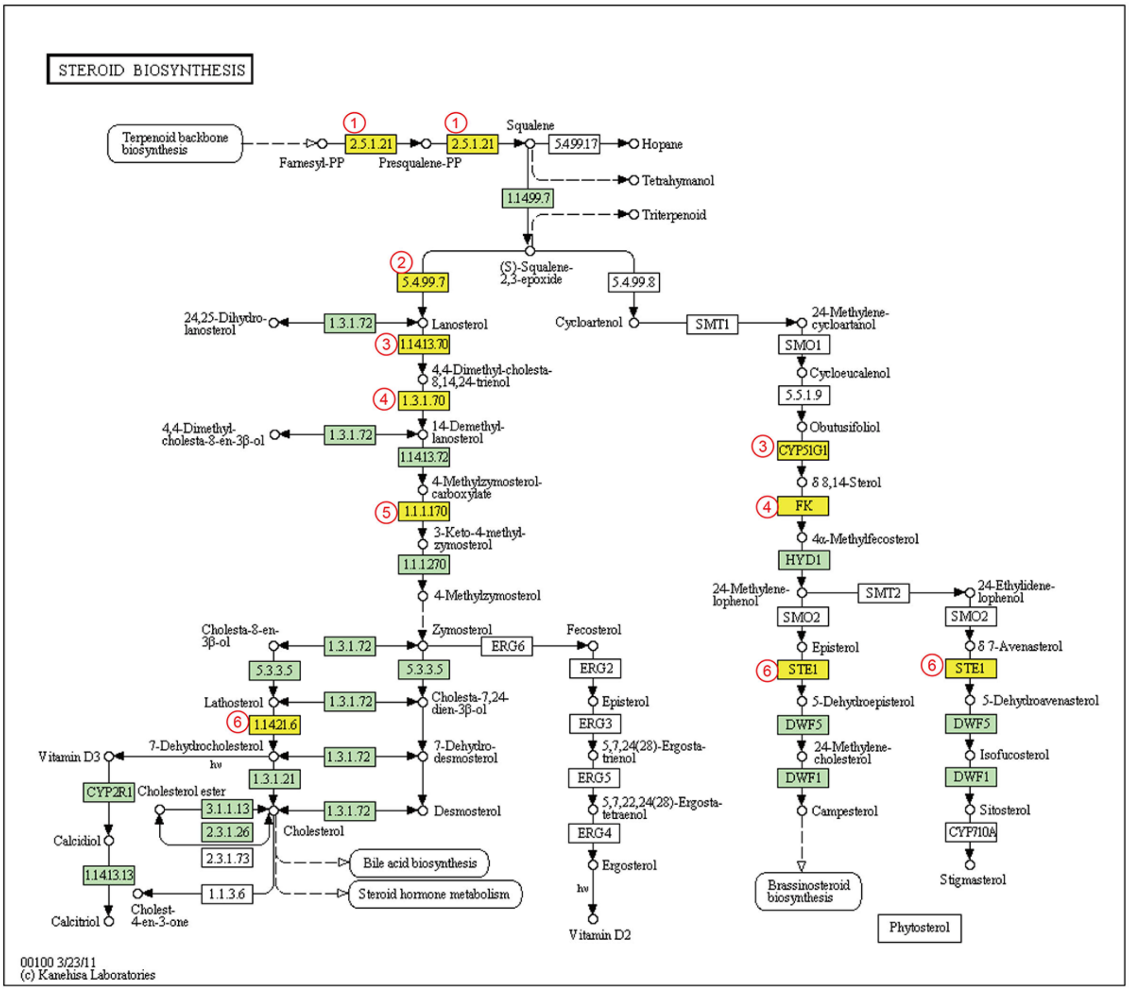

① farnesyl diphosphate farnesyl transferase 1 (Fdft1)
② lanosterol synthase(Lss)
③ cytochrome P450, family 51 (Cyp51)
④ transmembrane 7 superfamily member 2 (Tm7sf2)
⑤ NAD(P) dependent steroid dehydrogenase-like (Nsdhl)
⑥ sterol-C5-desaturase (fungal ERG3, delta-5-desaturase) homolog (S. cerevisae)(Sc5d)

Supplementary Figure S4. Hydrogen enhances the expression of a wide variety of steroid biosynthesis-related genes.

Differentially expressed genes identified in the KEEG pathway database related to steroid biosynthesis are shown. Genes for which the expression was significantly increased by H_2-water in the pathway are indicated in yellow.

Supplementary Figure S5

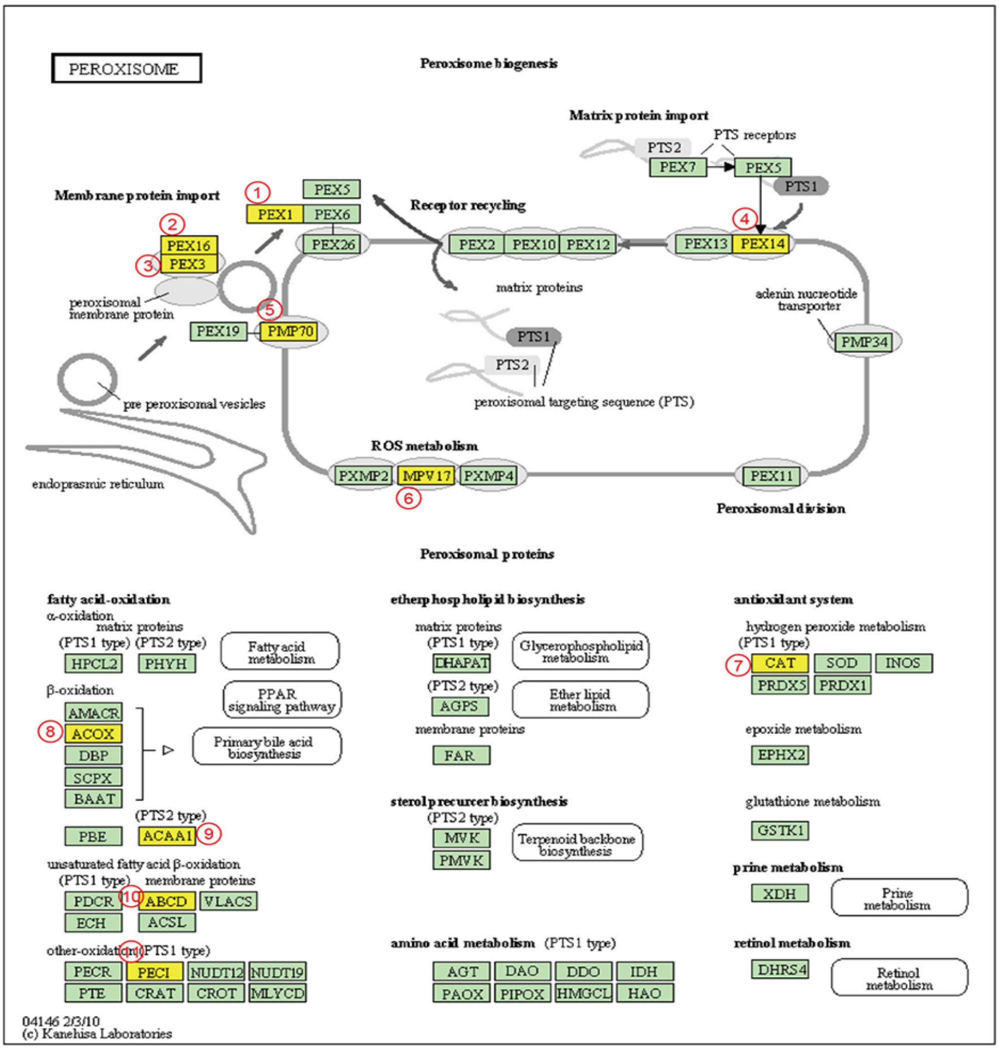

① peroxisomal biogenesis factor 1 (Pex1)
② peroxisomal biogenesis factor 16 (Pex16)
③ peroxisomal biogenesis factor 3(Pex3)
④ peroxisomal biogenesis factor 14 (Pex14)
⑤ ATP-binding cassette, sub-family D (ALD), member 3 (Abcd3)
⑥ Mpv17 transgene, kidney disease mutant-like(Mpv17l)
⑦ catalase (Cat)
⑧ acyl-coenzyme A oxidase 1, palmitoyl (Acox1)
⑨ acetyl-coenzyme A acyltransferase(Acaa1a, Acaa1b)
⑩ ATP-binding cassette, sub-family D (ALD), member 3 (Abcd3)
⑪ peroxisomal delta3, delta2-enoyl-Coenzyme A isomerase (Peci)

Supplementary Figure S5. Hydrogen enhances the expression of a wide variety of peroxisome-related genes.

Differentially expressed genes identified in the KEGG pathway database related to peroxisome are shown. Genes for which the expression was significantly increased by H_2-water in the pathway are indicated in yellow.

Supplementary Figure S6

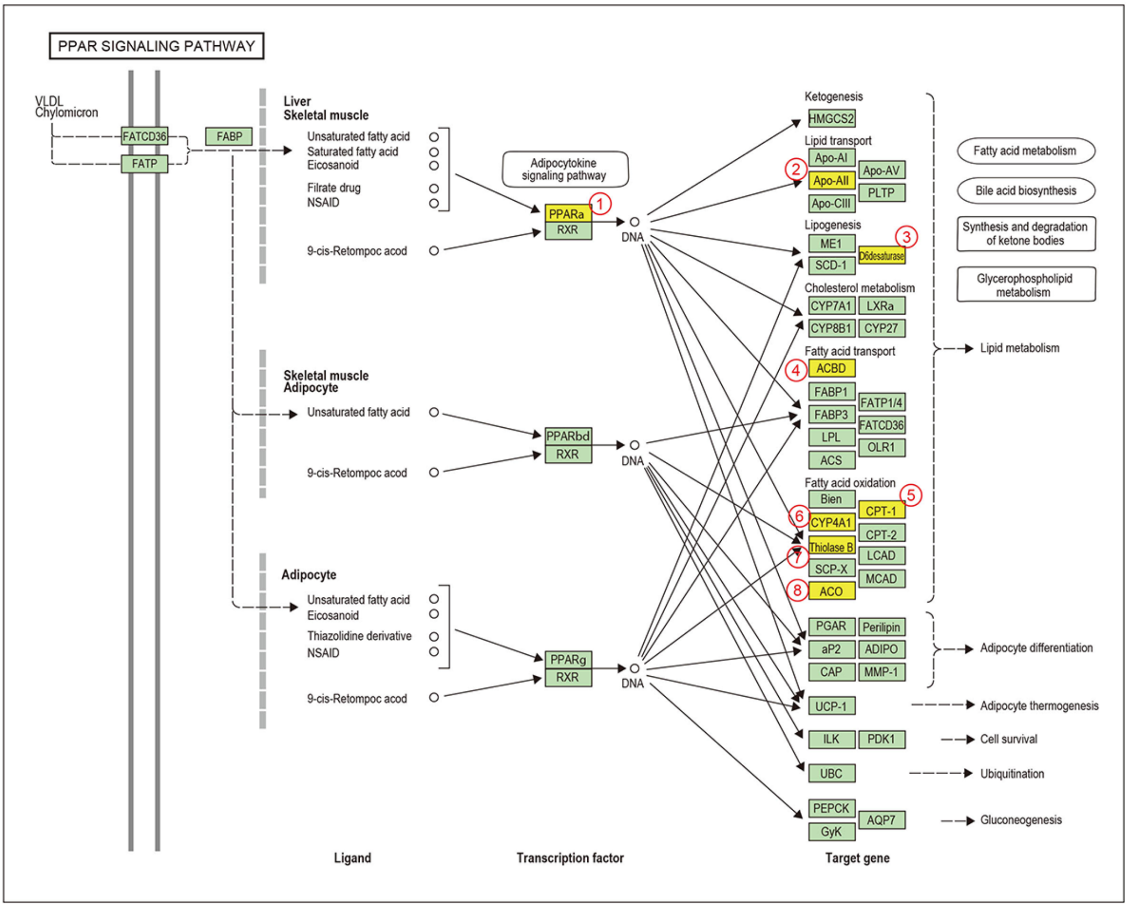

① peroxisome proliferator activated receptor alpha (Ppara)
② apolipoprotein A-II(Apoa2)
③ fatty acid desaturase 2 (Fads2)
④ diazepam binding inhibitor(Dbi)
⑤ carnitine palmitoyltransferase 1a, liver (Cpt1a)
⑥ cytochrome P450, family 4, subfamily a, polypeptide (Cyp4a32, Cyp4a10, Cyp4a31)
⑦ acetyl-coenzyme A acyltransferase(Acaa1a, Acaa1b)
⑧ acyl-coenzyme A oxidase 1, palmitoyl (Acox1)

Supplementary Figure S6. Hydrogen enhances the expression of a wide variety of PPAR signaling pathway-related genes.
Differentially expressed genes identified in the KEGG pathway database related to the PPAR signaling pathway are shown. Genes for which the expression was significantly increased by H_2-water in the pathway are indicated in yellow.

Supplementary Figure S7

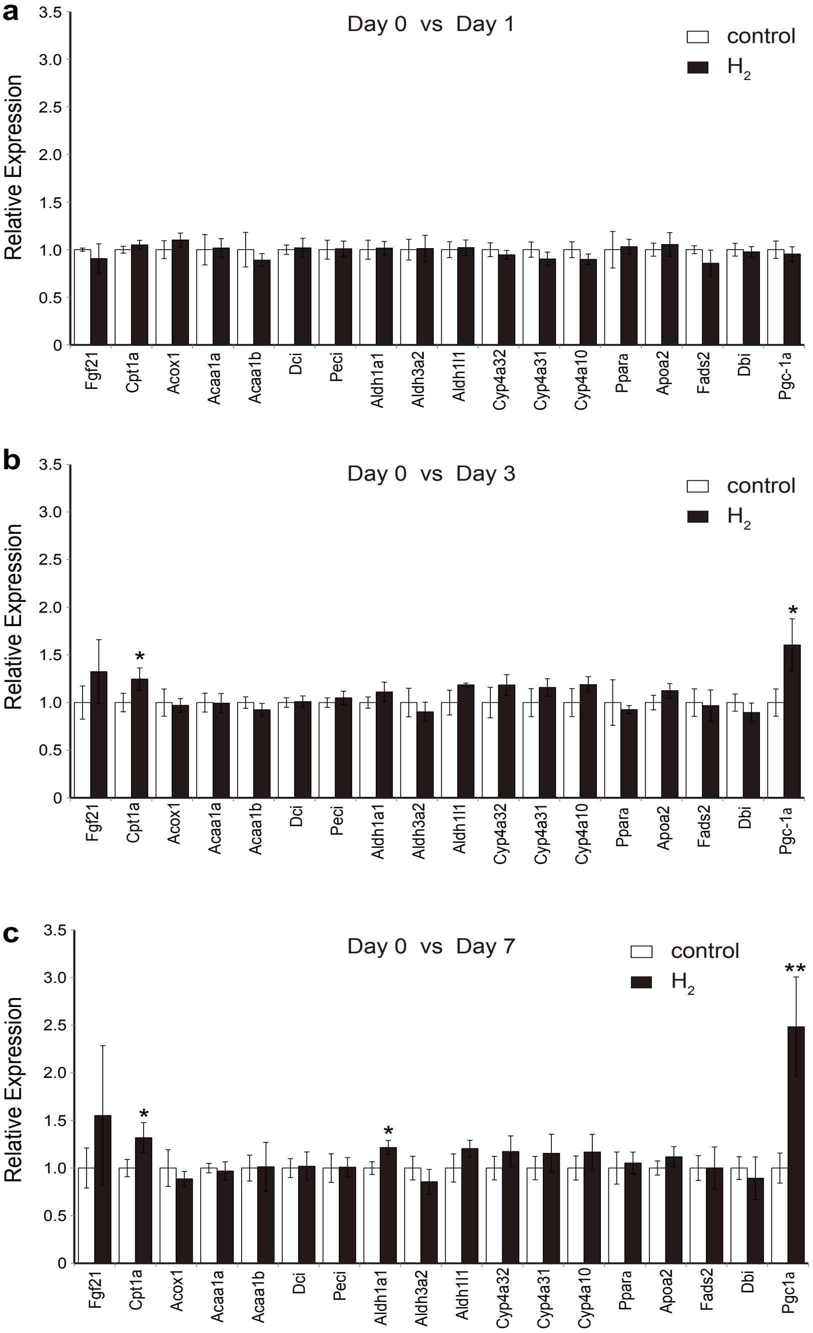

Supplementary Figure S7. Relative expression on DNA microarray of genes selected by the result of 2 weeks of consumption of H_2-water. *Db/db* mice were given 0.9 mg/kg MgH_2 for 0, 1, 3, and 7 days. Total RNA was prepared from the liver and DNA microarray analysis was performed. Relative signal intensities of genes selected by the result of 2 weeks of consumption of H_2-water are shown. Data are mean ± SD (n=3).

Supplementary Figure S8

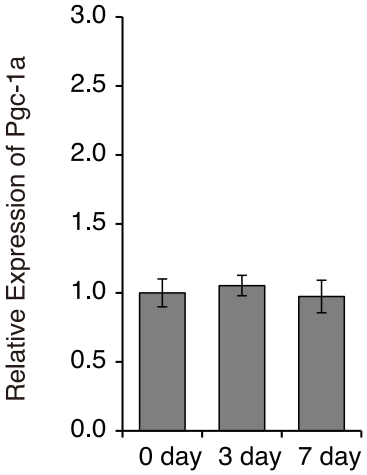

Supplementary Figure S8. Relative expression of the PGC-1α gene after the administration of MgH_2 in wild type.

C57BL/6 mice were given 0.9 mg/kg MgH_2 for 3 and 7 days. Total RNA was prepared from the liver, the expression of the PGC-1α gene was estimated by using quantitative RT-PCR analysis. Data are mean ± SD (n=9).

Supplementary Table S1 Nucleotide sequences of primer sets and TaqMan probes.

Gene	Forward primer	Reverse primer	TaqMan probe
Mouse			
Ppargc1a	ACCCTGCCATTGTTAAGACC	CTGCTGCTGTTCCTGTTTTC	CAACAGCAAAAGCCACAAAGACGTC
Fgf21	CCGCAGTCCAGAAAGTCTCCT	TCTGAAGCTGCAGGCCTCA	AGCTCTCTATGGATCGCCTCACTTTGATC C
Cpt1a	CTATGCGCTACTCGCTGAAG	AGACTCCAGGTACCTGCTCA	CTGCCTGTCCCAGCTGTCAAAGAT
Acox1	TTGTCCCTATCCGTGAGATT	AAACCATGGTCCCATATGTC	AAGCCTCTGCCAGGCATCACTGTT
Apoa2	CTGCTGGTCACCATCTGTAG	TAGTTCCTGCTGACCTGACA	CCAGGCCAAGGCATACTTTGAGAAG
Aldh1a1	ATGGTTTAGCAGCAGGACTC	AAAGACCATGTTCACCCAGT	TGCCCCTTCGGTGGATTCAA
Ppara	TTCAGAAGAAGAACCGGAAC	CTTTCAGGTCGTGTTCACAG	CGGGATGTCACACAATGCAATTCGC
Acaa1a	GCATCCCAGAGACTGTACCT	GTCATCCCCATAGGAGTCAG	TGCTGGAGAGTGAGAAAGCCAGAGA
GAPDH	CATCACTGCCACCCAGAAGA	ATGTTCTGGGCAGCC	TGGATGGCCCCTCTGGAAAGCTG
Human (HepG2 cell)			
Pgc1a	GATGGCCTGTTTGATGACAG	TTTGGGTGGTGACACAGAAT	TACCCTTGGGATGGCACGCA
GAPDH	GGGAAGGTGAAGGTCGGA	GCAGCCCTGGTGACCAG	CAACGGATTTGGTCGTATTGGGCG

Complementary DNA was generated by SuperScript II Reverse Transcriptase (Invitrogen, Waltham, MA, USA) from RNA samples that were used for microarray analysis. cDNA was analyzed by quantitative PCR using Thermal Cycler Dice Real Time System TP800 (TAKARA BIO INC., Otsu, Shiga, Japan). All samples were normalized to glyceraldehyde 3-phosphate dehydrogenase (GAPDH) expression. Primer and probe sequences for each PCR are shown.
